# A subgroup of plant aquaporins facilitate the bi-directional diffusion of As(OH)_3 _and Sb(OH)_3 _across membranes

**DOI:** 10.1186/1741-7007-6-26

**Published:** 2008-06-10

**Authors:** Gerd P Bienert, Michael Thorsen, Manuela D Schüssler, Henrik R Nilsson, Annemarie Wagner, Markus J Tamás, Thomas P Jahn

**Affiliations:** 1 Department of Agricultural Sciences, Faculty of Life Sciences, University of Copenhagen, Frederiksberg C, Denmark; 2 Department of Cell and Molecular Biology/Microbiology, University of Gothenburg, Göteborg, Sweden; 3 Department of Plant and Environmental Sciences, University of Gothenburg, Göteborg, Sweden; 4 Department of Chemistry, Atmospheric Science, University of Gothenburg, Göteborg, Sweden; 5 Current Address: UCL Université catholique de Louvain, Unité de biochemie physiologique, Croix du Sud 5/15, 1348 Louvain-la-Neuve, Belgium

## Abstract

**Background:**

Arsenic is a toxic and highly abundant metalloid that endangers human health through drinking water and the food chain. The most common forms of arsenic in the environment are arsenate (As(V)) and arsenite (As(III)). As(V) is a non-functional phosphate analog that enters the food chain via plant phosphate transporters. Inside cells, As(V) becomes reduced to As(III) for subsequent extrusion or compartmentation. Although much is known about As(III) transport and handling in microbes and mammals, the transport systems for As(III) have not yet been characterized in plants.

**Results:**

Here we show that the Nodulin26-like Intrinsic Proteins (NIPs) AtNIP5;1 and AtNIP6;1 from *Arabidopsis thaliana*, OsNIP2;1 and OsNIP3;2 from *Oryza sativa*, and LjNIP5;1 and LjNIP6;1 from *Lotus japonicus *are bi-directional As(III) channels. Expression of these NIPs sensitized yeast cells to As(III) and antimonite (Sb(III)), and direct transport assays confirmed their ability to facilitate As(III) transport across cell membranes. On medium containing As(V), expression of the same NIPs improved yeast growth, probably due to increased As(III) efflux. Our data furthermore provide evidence that NIPs can discriminate between highly similar substrates and that they may have differential preferences in the direction of transport. A subgroup of As(III) permeable channels that group together in a phylogenetic tree required N-terminal truncation for functional expression in yeast.

**Conclusion:**

This is the first molecular identification of plant As(III) transport systems and we propose that metalloid transport through NIPs is a conserved and ancient feature. Our observations are potentially of great importance for improved remediation and tolerance of plants, and may provide a key to the development of low arsenic crops for food production.

## Background

Arsenic is widespread in the Earth's crust and is highly available in the biosphere. Arsenic is acutely toxic to all organisms and is rated as a group I human carcinogen by the International Agency for Research of Cancer [1]. Due to its high bioavailability and toxicity, arsenic is considered a global health hazard. Contaminated drinking water is the main source of arsenic intake in several parts of the world. In Argentina, Australia, Bangladesh, Chile, China, Hungary, India, Mexico, Peru, Thailand, and the United States of America, arsenic concentrations higher than the permissible levels have been reported and negative effects on human health have been documented [2]. The second largest source of arsenic for humans is through ingestion of food that has accumulated arsenic due to the irrigation of crop plants with polluted water [3]. In Argentina, arsenic concentrations of 300–400 parts per billion (ppb) were measured in soup and maize porridge [4] in villages where the drinking water was about 200 ppb [4,5], a concentration that is already considerably higher than the upper limit for drinking water of 10 ppb as recommended by the World Health Organization [2]. It is estimated that, worldwide, 1–24% of the tolerable intake of inorganic arsenic is solely due to the consumption of arsenic-containing rice [6]. Consequently, a detailed understanding of arsenic uptake and detoxification in plants is of great interest and is important for strategies in plant biotechnology for designing safer crops as well as for generating plants for the use in phytoremediation [7].

Arsenate (As(V)) is the prevailing arsenic species in aerated soils. As(V) is a non-functional phosphate analog that is taken up via plant phosphate transporters [8]. Inside the plant, most of the As(V) is reduced to arsenite (As(III)) via arsenate reductases [9,10]. As(III) reacts with sulfhydryl groups in cysteine residues and the imidazolium nitrogen in histidine residues leading to interference with general protein function [11]. To control As(III) levels in the cytoplasm, As(III) can be conjugated to phytochelatins [12] and probably sequestered into vacuoles by ABC transporters [13]. Most of the vacuolar arsenic however is inorganic As(III) [14,15]. In *Arabidopsis*, As(III) constitutes up to 70% of total arsenic and appears to be the main transported species [16]. Transporters involved in As(III) distribution and sequestration at the cellular level are still unknown. At the whole-plant level, arsenic can be transported from root to shoot via the vascular system. In the xylem sap, the predominant arsenic species are inorganic As(III) and As(V) [17,18] but methylated species are also present at low concentrations [17,19].

Recently, aquaglyceroporins from microbes and mammals have been shown to facilitate the movement of As(III) and the related metalloid antimonite (Sb(III)) across cell membranes (as reviewed in [20]). Moreover, in *Arabidopsis thaliana *and *Oryza sativa *(rice), members of the Nodulin26-like Intrinsic Protein (NIP) subfamily of plant aquaporins, AtNIP5;1 and OsNIP2;1, have been localized to the plasma membrane in planta and were shown to play physiologically important roles in the uptake of the nutritionally important metalloids boron and silicon, respectively [21,22]. Finally, competition studies for the uptake of As(III) with glycerol in rice led to the suggestion that As(III) is also transported through members of the aquaporin family in plants [23]. Based on these observations and the fact that members of the NIP subfamily are believed to be the functional equivalents of aquaglyceroporins, we hypothesized that NIPs may be As(III) transporters. To test this prediction directly, we cloned various NIP isoforms from *A. thaliana*, *Lotus japonicus*, and *O. sativa *and expressed them in *Saccharomyces cerevisiae *(budding yeast) as a heterologous test system for metalloid transport. Our data demonstrate that the capacity to channel As(III) and Sb(III) is conserved in the NIPII subgroup in plants. Expression of specific NIPs was found to sensitize yeast cells to As(III) and Sb(III) and caused increased As(III) uptake into yeast. On medium containing As(V), expression of the same NIP isoforms improved growth, probably due to increased efflux of As(III) accumulating in the yeast cells upon As(V) reduction. Strikingly, a number of NIP isoforms proved particularly potent in releasing As(III) from yeast cells exposed to As(V), suggesting that specific NIPs could play a role in As(III) detoxification in plants.

## Results

### Expression of specific NIPs from *Arabidopsis *sensitizes yeast Δ*fps1 *to As(III) and Sb(III)

**Figure 1:**
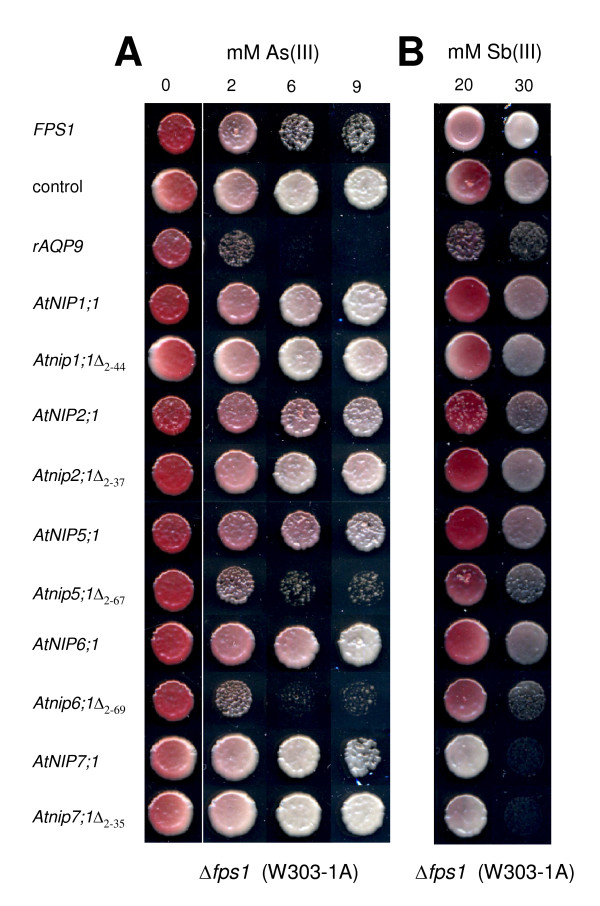
**Yeast expressing specific *Arabidopsis *aquaporin homologs display sensitivity to arsenite (As(III)) and antimonite (Sb(III))**. Δ*fps1 *(background W303-1A) cells were transformed with the empty vector pYES2.1 (control) or aquaporin homologs *rAQP9*, *ScFPSI*, *AtNIP1;1*, *Atnip1;1*Δ_2–44_, *AtNIP2;1*, *Atnip2;1*Δ_2–37_, *AtNIP5;1*, *Atnip5;1*Δ_2–67_, *AtNIP6;1*, *Atnip6;1*Δ_2–69_, *AtNIP7;1 *or *Atnip7;1*Δ_2–35 _and spotted at an A_600 _of 0.01 on medium containing various concentrations of (A) As(III) or (B) Sb(III) as indicated. Growth was recorded after 5 days at 30°C. All data were duplicated in at least two independent experiments with consistent results.

**Figure 2:**
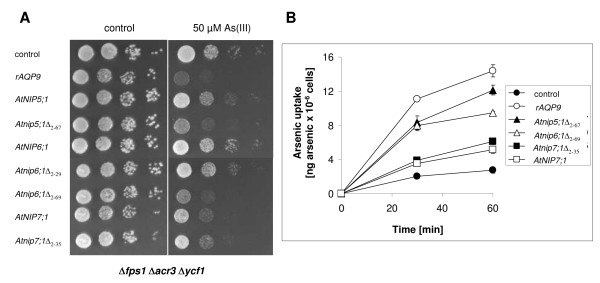
***Arabidopsis *aquaporins mediate arsenic influx into yeast as monitored by growth and transport assays**. (A) Growth assay. Plasmids expressing aquaporin homologs *rAQP9*, *AtNIP5;1*, *Atnip5;1*Δ_2–67_, *AtNIP6;1*, *Atnip6;1*Δ_2–29_, *Atnip6;1*Δ_2–69_, *AtNIP7;1 *or *Atnip7;1Δ*_2–35 _as well as an empty vector control (pYES2.1) were transformed into Δ*fps1 *Δ*acr3 *Δ*ycf1*. The transformants were grown in liquid medium and 10-fold serial dilutions of the cultures were spotted on agar plates with or without arsenite (As(III)). Growth was monitored after 2 to 3 days at 30°C. (B) As(III) uptake. The *Δfps1 Δacr3 Δycf1 *mutant harboring the empty pYES2.1 plasmid or plasmids expressing the indicated proteins were exposed to As(III) and intracellular arsenic levels were determined as described in Methods. The data shown represent the average of at least three independent experiments and the error bars represent the standard deviation.

To test whether plant aquaporin homologs transport As(III) and Sb(III), *AtNIP1;1*, *AtNIP2;1*, *AtNIP5;1*, *AtNIP6;1 *and *AtNIP7;1 *were heterologously expressed in the aquaglyceroporin deficient *Δfps1 *yeast mutant. As As(III) and Sb(III) enter yeast cells through Fps1p [24,25], *Δfps1 *cells are characterized by low influx and enhanced resistance towards these metalloids. As positive controls, we used *AQP9 *from rat (*rAQP9*) and *ScFPS1 *that reverse metalloid tolerance of *Δfps1 *cells [24,26]. Yeast transformants were plated on medium containing various concentrations of As(III) or Sb(III) and growth was scored (Figure 1). Growth of yeast expressing *rAQP9 *was reduced on medium containing 2 mM As(III) and completely repressed in the presence of 6 mM As(III), whereas the control yeast transformed with the empty vector grew well at concentrations up to 9 mM As(III). Yeast transformed with *ScFPS1 *showed reduced growth at 6 and 9 mM As(III) (Figure 1A). On medium containing Sb(III), growth of yeast transformed with *rAQP9 *was strongly affected whereas the control grew well at 30 mM Sb(III) (Figure 1B). Yeast cells expressing *AtNIP7;1 *were strongly Sb(III)-sensitive (Figure 1B) but only slightly As(III)-sensitive (Figures 1 and 2A). In contrast, none of the other plant aquaporin isoforms appeared to sensitize yeast cells to As(III) or Sb(III) (Figure 1). It was previously reported that, for functional expression of certain NIP isoforms in yeast, it is necessary to truncate parts of their hydrophilic N-terminal domain [27]. Therefore, we genetically engineered N-terminally truncated versions of several NIPs including *Atnip1;1*Δ_2–44_, *Atnip2;1*Δ_2–37_, *Atnip5;1*Δ_2–67_, *Atnip6;1*Δ_2–69 _and *Atnip7;1*Δ_2–35_. Importantly, yeast expressing the truncated *Atnip5;1*Δ_2–67 _and *Atnip6;1*Δ_2–69 _showed strong sensitivity towards As(III) (Figure 1A) whereas growth of these cells was only slightly affected by Sb(III) (Figure 1B). On the other hand, expression of *Atnip7;1*Δ_2–35 _resulted in a very similar growth phenotype as expression of the full length *AtNIP7;1*, both on As(III)- and Sb(III)-containing plates (Figures 1 and 2A). *AtNIP1;1 *has previously been functionally expressed in yeast as a full-length protein [28]. Neither full length *AtNIP1;1 *nor truncated *Atnip1;1*Δ_2–44 _affected metalloid sensitivity (Figure 1). Similarly, expression of *AtNIP2;1 *or *Atnip2;1*Δ_2–37 _(Figure 1), or a truncated version of AtNIP6;1 lacking only 28 aa of the N-terminal domain (*Atnip6;1*Δ_2–29_), had no impact on metalloid sensitivity (discussed in the following).

### Expression of specific NIPs from *Arabidopsis *sensitizes the Δ*fps1 *Δ*acr3 *Δ*ycf1 *to As(III) and facilitates As(III) uptake

Yeast cells are equipped with highly sophisticated arsenic detoxification mechanisms including the arsenate reductase Acr2p, the plasma membrane localized As(III) extrusion transporter Acr3p, and the vacuolar ABC transporter Ycf1p (as reviewed in [29]). Consequently, yeast is relatively resistant towards externally supplied arsenic and addition of millimolar concentrations of As(III) to the medium was needed to score growth reduction of Δ*fps1 *cells expressing *Atnip5;1*Δ_2–67_, *Atnip6;1*Δ_2–69_, *AtNIP7;1 *and *Atnip7;1*Δ_2–35 _(see Figure 1). Such a concentration range is rather rare in the plant's environment. In addition, the resulting high-concentration gradient across the yeast plasma membrane could force As(III) and Sb(III) to permeate the channels. Therefore, we expressed specific aquaporin isoforms in the *Δfps1 Δacr3 Δycf1 *yeast mutant that is defective in As(III) detoxification and displays enhanced As(III) sensitivity [24]. *Δfps1 Δacr3 Δycf1 *cells expressing *rAQP9*, *Atnip5;1*Δ_2–67_, *Atnip6;1*Δ_2–69_, *AtNIP7;1 *and *Atnip7;1*Δ_2–35 _displayed increased As(III) sensitivity compared with the empty vector control whereas cells expressing full-length *AtNIP5;1 *and *AtNIP6;1 *were not different from the control (Figure 2A).

We next tested the ability of these NIPs to transport As(III) in direct uptake measurements. The *Δfps1 Δacr3 Δycf1 *mutant has a low level of As(III) influx (Figure 2B). Expression of *rAQP9*, *Atnip5;1*Δ_2–67_, *Atnip6;1*Δ_2–69_, *AtNIP7;1 *and *Atnip7;1*Δ_2–35 _led to a significant increase in As(III) uptake after 30 and 60 minutes of exposure. The amount of As(III) taken up by the different transformants corresponded well with the degree of As(III) sensitivity seen when growing the respective transformant on As(III)-containing medium (*rAQP9 *> *Atnip5;1*Δ_2–67 _> *Atnip6;1*Δ_2–69 _> *Atnip7;1*Δ_2–35 _≈ *AtNIP7;1*; see Figure 2A and 2B). Moreover, we note that N-terminal truncation of AtNIP7;1 only affected As(III) transport to a minor extent (Figure 2B), which is in line with the observed phenotypes (Figure 2A). Finally, the relatively low level of As(III) influx into cells expressing full-length or truncated AtNIP7;1 is consistent with the observation that sensitivity is only clearly visible in the sensitized *Δfps1 Δacr3 Δycf1 *mutant.

### Expression of specific NIPs from *Arabidopsis *improves growth of Δ*fps1 *Δ*acr3 *Δ*ycf1 *on medium containing As(V)

**Figure 3:**
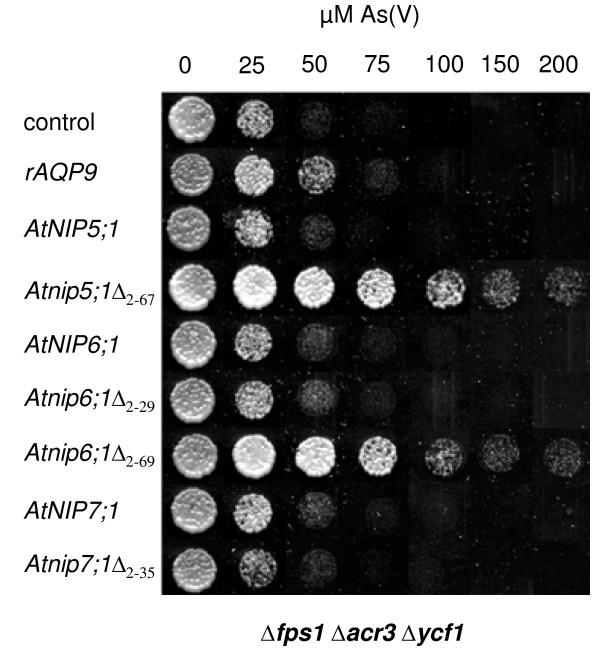
**Expression of *Arabidopsis *aquaporin homologs improves yeast growth in the presence of arsenate (As(V))**. Δ*fps1 *Δ*acr3 *Δ*ycf1 *transformed with the empty vector pYES2.1 (control) or the indicated aquaporin homologs were spotted at an A_600 _of 0.01 on medium containing various concentrations of As(V) as indicated. Growth was recorded after 6 days at 30°C.

As(V) enters yeast cells via phosphate transporters. To avoid interference with phosphate assimilation and metabolism, yeast, like other organisms, reduces As(V) to the more mobile As(III) for subsequent extrusion and sequestration, as reviewed in [29]. The *Δfps1 Δacr3 Δycf1 *yeast mutant lacks transport systems for As(III) and, as the result of the action of Acr2p, accumulates As(III) in the presence of externally supplied As(V). *Δfps1 Δacr3 Δycf1 *is therefore more sensitive towards externally supplied As(V) [24]. When transformed with the empty vector, growth of *Δfps1 Δacr3 Δycf1 *was severely affected by 50 μM As(V) (Figure 3). Similar growth was recorded for cells expressing full-length *AtNIP5;1 *and *AtNIP6;1*, which also did not facilitate As(III) influx (see Figure 1). In contrast, the expression of *rAQP9*, *Atnip5;1*Δ_2–67 _and *Atnip6;1*Δ_2–69 _rendered cells more resistant to externally supplied As(V) (Figure 3). Cells expressing *rAQP9 *grew slightly better on As(V) than control cells whereas cells expressing *Atnip5;1*Δ_2–67 _and *Atnip6;1*Δ_2–69 _were able to grow in the presence of up to 0.2 mM As(V). The fact that certain NIPs can improve growth in the presence of As(V) strongly suggests that they mediate As(III) efflux and supports the notion that these proteins are bi-directional channels. Expressing *AtNIP7;1 *did not improve As(V) resistance compared with the empty vector control. Strikingly, although expression of *rAQP9 *resulted in more severe growth inhibition on medium containing As(III) than expression of *Atnip5;1*Δ_2–67 _and *Atnip6;1*Δ_2–69 _(Figure 2A), yeast transformed with *Atnip5;1*Δ_2–67 _and *Atnip6;1*Δ_2–69 _grew much better on medium containing As(V) than those transformed with *rAQP9 *(Figure 3). This observation suggests that AtNIP5;1, AtNIP6;1 and rAQP9 may have different preferences regarding the direction of transport.

### The capacity of NIP homologs to transport As(III) and Sb(III) is conserved across plant species

**Figure 4:**
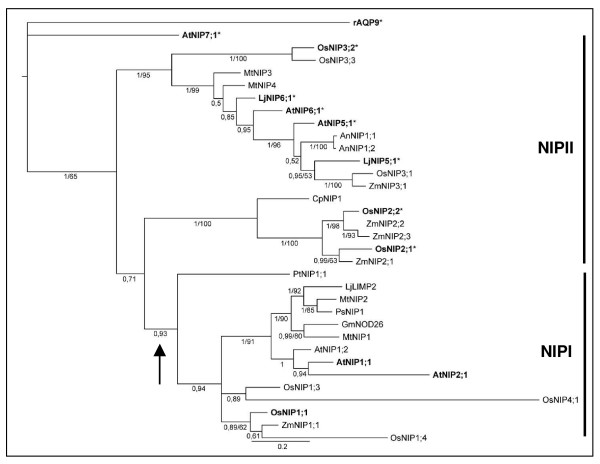
**The consensus phylogram from the Bayesian phylogenetic inference of 33 NIP isoforms from various plant species**. Rat AQP9 (rAQP9) was used as an out-group. Support values are given below the branches as Bayesian posterior probabilities or jackknife values from the parsimony analysis (cut-off threshold corresponding to 50% for both); the latter is specified as applicable. The scale given below the phylogram refers to expected changes per site. The NIP proteins tested in this paper are in bold and those that transport As(III) are marked with an asterisk (*). The NIPI and NIPII subgroups are indicated with a vertical bar and an arrow indicates the hypothetical point in evolution where the NIPI subgroup may have lost the capacity to transport metalloids.

**Figure 5:**
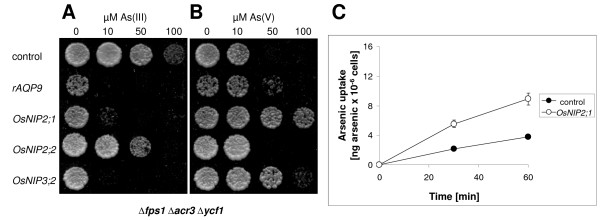
**Growth of yeast cells expressing aquaporin homologs from *Oryza sativa***. Δ*fps1 *Δ*acr3 *Δ*ycf1 *transformed with the empty vector pYES2.1 (control) or *rAQP9*, *OsNIP2;1*, *OsNIP2;2 *or *OsNIP3;2 *were spotted at an A_600 _of 0.01 on medium containing various concentrations of (A) As(III) or (B) As(V) as indicated. Growth was recorded after 4 days at 30°C. (C) OsNIP2;1 mediates uptake of As(III) into yeast. As(III) transport was measured as described in Methods.

To gain more functional understanding of the NIP proteins, we first performed a phylogenetic analysis including 33 NIP homologs from various species, using rat AQP9 as an out-group (Figure 4). The phylogenetic analyses support, and refine further, the subdivision of the NIP subfamily of the aquaporin superfamily into the two subgroups NIPI and NIPII [30-33]. Together, the phylogenetic analysis, transport assays, and phenotypic data presented above, suggested that the capacity to facilitate diffusion of As(III) and Sb(III) across membranes is associated with members of the NIPII subgroup. To substantiate our hypothesis, one isoform of subgroup I (*OsNIP1;1*) and three isoforms of subgroup II (*OsNIP2;1*, *OsNIP2;2 *and *OsNIP3;2*) from rice were expressed in yeast and tested for their permeability towards As(III). Expression of *OsNIP2;1 *and *OsNIP3;2 *sensitized yeast to As(III) (Figure 5A) and Sb(III) (data not shown), whereas their expression improved growth of *Δfps1 Δacr3 Δycf1 *on As(V)-containing medium (Figure 5B). Expression of *OsNIP2;2 *produced moderate As(III) and Sb(III) sensitivities (Figure 5A and data not shown). In contrast, *OsNIP1;1 *expression did not affect growth at all (data not shown). Finally, direct transport assays confirmed that the increased sensitivity of cells expressing *OsNIP2;1 *towards As(III) was the result of increased influx into those cells (Figure 5C).

### Lack of competitive uptake between As(III) and B(OH)_3 _or Si(OH)_4 _into yeast cells expressing *AtNIP5;1 *and *OsNIP2;1*

OsNIP2;1 and AtNIP5;1 were earlier identified as physiologically important Si(OH)_4 _and B(OH)_3 _channels in rice [22] and *Arabidopsis *[21], respectively. When expressed in oocytes, AtNIP5;1 mediated the uptake of B(OH)_3 _and OsNIP2;1 mediated the uptake of Si(OH)_4_. Here we demonstrated that As(III) is an additional and common substrate for both proteins. To address whether there is selectivity for the uptake of one metalloid over another, we exposed yeast cells expressing *OsNIP2;1 *to As(III) alone or together with various concentrations of Si(OH)_4_. However, there was no significant difference in the uptake of As(III), even at a 10-fold higher concentration of Si(OH)_4 _over As(III) (data not shown). Similarly, no significant difference in As(III) uptake through Atnip5;1Δ_2–67 _could be measured when yeast cells were exposed to both As(III) and B(OH)_3 _even at a 10-fold higher concentration of B(OH)_3 _over As(III) (data not shown). Higher concentrations of Si(OH)_4 _in the uptake assay cannot be used because of polymerization of monosilic acid to polysilicic acid, and higher concentration of B(OH)_3 _will lower the pH of the medium. Thus, at physiologically relevant concentrations of Si(OH)_4 _and B(OH)_3_, no measurable competition for the uptake of As(III) could be detected.

### Proper functional expression of some NIP isoforms in yeast requires deletion of the N-terminal hydrophilic domain

**Figure 6:**
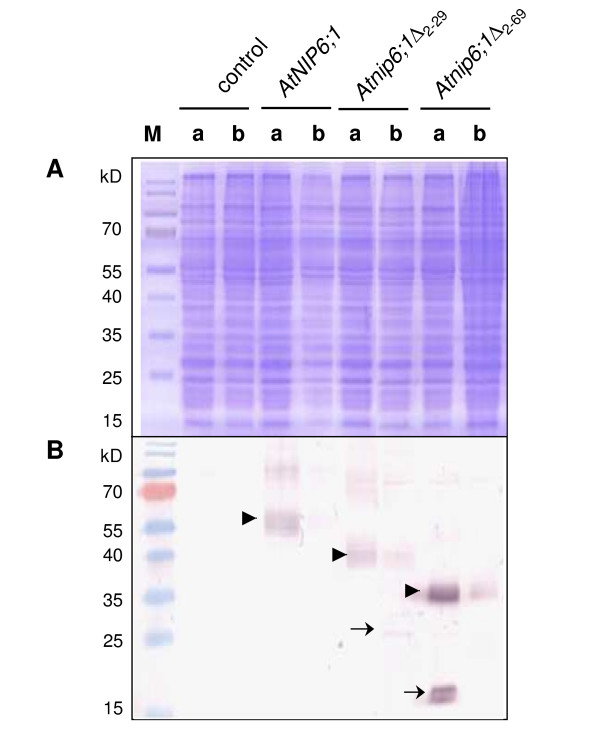
**Detection by Western blotting of AtNIP6;1, Atnip6;1Δ_2–29 _and Atnip6;1Δ_2–69 _expressed in yeast**. We ran 20 μg of microsomal membrane proteins from (a) Δ*fps1 *and (b) Δ*fps1 *Δ*acr3 *Δ*ycf1 *transformed with the empty vector or *AtNIP6;1*, *Atnip6;1*Δ_2–29 _or *Atnip6;1*Δ_2–69 _on 12% SDS polyacrylamid gels. Proteins were (A) stained with commassie brilliant blue or (B) transferred to nitrocellulose membrane for probing with an anti-AtNIP6;1 specific antibody. M, PageRuler™ Prestained Protein Ladder (Fermentas). The arrows point towards bands that probably represent monomeric forms of *Atnip6;1*Δ_2–29 _and *Atnip6;1*Δ_2–69_. Arrowheads point towards bands that probably represent oligomeric forms of the proteins.

As shown above, N-terminal truncation allows functional expression of certain NIPs in yeast. To test whether truncation is necessary for proper expression of NIPs in yeast, we extracted microsomal membranes from Δ*fps1 *and Δ*fps1 *Δ*acr3 *Δ*ycf1 *cells transformed with *AtNIP6;1*, *Atnip6;1*Δ_2–29 _and *Atnip6;1*Δ_2–69 _and probed the extracts by Western-blot analysis using an anti-AtNIP6;1 specific antibody (Figure 6). The antibody was raised against a peptide corresponding to a sequence in the C-terminal end of the protein (Per Kjellbom and Urban Johanson, personal communication). Therefore, the signal on a Western blot should reflect the relative abundance of full-length and truncated versions of AtNIP6;1 in these extracts. In microsomal membranes from yeast transformed with full-length *AtNIP6;1 *or both truncated versions, a clear signal could be detected between 35 and 55 kD, which might represent the oligomeric forms of the respective protein (Figure 6, arrowheads). The bands corresponding to the truncated forms of AtNIP6;1 migrated faster, as expected from the lower molecular weight due to deletion of the N-terminal 28 and 68 aa, respectively and the monomeric forms were also detected (Figure 6, arrows). Truncation of aa 2–29 had little effect on protein levels, whereas truncation of aa 2–69 resulted in higher amount compared with full length *AtNIP6;1*. We note that the various AtNIP6;1 forms did not migrate according to their molecular size, a phenomenon commonly observed for proteins of the aquaporin family [34,35]. The amount of the heterologous proteins was generally lower in the triple mutant Δ*fps1 *Δ*acr3 *Δ*ycf1 *than in Δ*fps1*.

Interestingly, the necessity to remove part of the N-terminal domain does not seem to apply to all members of the NIPII subgroup, as *AtNIP7;1*, *OsNIP3;2 *and *OsNIP2;1 *could be functionally expressed in yeast as full-length proteins (Figures 2 and 5). We therefore hypothesized that the necessity to truncate the N-terminal domain for functional expression in yeast applies to a subpopulation within the NIPII subgroup.

**Figure 7:**
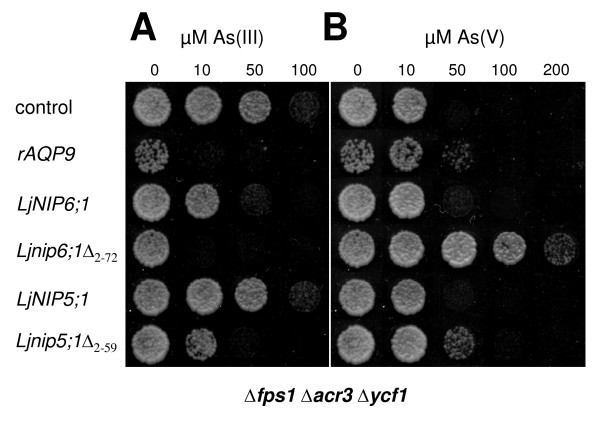
**N-terminal truncation is required for functional expression in yeast of specific *Lotus japonicus *aquaporin homologs**. Δ*fps1 *Δ*acr3 *Δ*ycf1 *transformed with the empty vector pYES2.1 (control) or *rAQP9*, *LjNIP5;1*, *Ljnip5;1*Δ_2–59_, *LjNIP6;1 *or *Ljnip6;1*Δ_2–72 _were spotted at an A_600 _of 0.01 on medium containing various concentrations of (A) As(III) or (B) As(V) as indicated. Growth was recorded after 4 days at 30°C.

To substantiate this notion, we cloned two additional NIPII homologs from the legume *L. japonicus*. Primers were designed according to Expressed Sequence Tag (EST) sequences of the N- and the C-termini of probable major intrinsic proteins of *L. japonicus *from the databases [36]. According to their close sequence similarity to *Arabidopsis *AtNIP5;1 and AtNIP6;1 (Figure 4) and because of a missing nomenclature for aquaporins in *L. japonicus*, we named them LjNIP5;1 and LjNIP6;1. The full-length coding sequences were submitted to GenBank [37] and are available under accession numbers EU294214 and EU294215, respectively. Expression of the N-terminally truncated versions of both LjNIP5;1 and LjNIP6;1 (*Ljnip5;1*Δ_2–72 _and *Ljnip6;1*Δ_2–59_) drastically sensitized *Δfps1 Δacr3 Δycf1 *cells to As(III) whereas expression of the corresponding full length clones did not (*LjNIP5;1*) or only to a moderate extent (*LjNIP6;1*) (Figure 7A). The truncated versions also sensitized cells to Sb(III) (data not shown). When As(V) was supplied to the growth medium, the expression of *Lotus Ljnip5;1*Δ_2–72 _and *Ljnip6;1*Δ_2–59 _increased growth compared with control cells transformed with the empty vector (Figure 7B). Hence, truncating the N-terminus of *LjNIP*s resulted in As(III) transport, similar to what was observed for *AtNIP5;1 *and *AtNIP6;1*.

## Discussion and conclusion

Aquaglyceroporins from bacteria, yeast, protozoa and mammals have been shown to facilitate transport of the metalloids As(III) and Sb(III) across cell membranes (as reviewed in [20]). Here, we demonstrate that members of the NIP subfamily of plant aquaporins, which are believed to be the functional equivalents to microbial and mammalian aquaglyceroporins, mediate As(III) and Sb(III) transport when expressed in yeast. We conclude that the capacity of aquaporins to transport As(III) and Sb(III) is also well conserved in plants.

It has been reported that truncating parts of the hydrophilic N-terminal domain of certain NIPs is necessary to achieve functional expression in yeast and that the N-terminus is not required for the transport activity itself [27]. Our data support these findings as N-terminal truncation of AtNIP5;1 and AtNIP6;1 was necessary for functional expression in yeast (Figures 1, 2 and 3). On the other hand, truncation of AtNIP7;1 had little effect on its transport capacity (Figures 1, 2 and 3), supporting the interpretation that the hydrophilic N-terminal domain is not important for basic channel function. Interestingly, the necessity to remove part of the N-terminal domain does not apply to all members of the NIPII subgroup, as AtNIP7;1, OsNIP3;2 and OsNIP2;1 could be functionally expressed in yeast as full-length proteins (Figures 1, 2, 3 and 5). This is in accordance with data from functional expression of another full-length NIPII isoform from *Cucurbita pepo *(CpNIP) in yeast [38]. Hence, the necessity to truncate the N-terminal domain for functional expression in yeast appears to apply to a subpopulation within the NIPII subgroup.

In contrast to yeast, *AtNIP5;1 *and *AtNIP6;1 *were successfully expressed in *Xenopus laevis *oocytes as full-length proteins [21,31]. In fact, it is not known why truncation is required for functional expression in yeast. Our Western-blot analysis of AtNIP6;1 suggests that the physical expression could be part of the reason; truncation of aa 2–69 resulted in increased protein levels in yeast membranes when compared with full-length AtNIP6;1. We can, however, not exclude that other reasons may contribute to this phenomenon. Deletion of the N-terminal domain could influence the targeting of the protein in the yeast secretory pathway. Alternatively, the N-terminal domain could function in gating the channel and components of a putative post-translational regulation mechanism displacing an inhibitory domain may be absent in yeast. A function of the N-terminal domain in gating has been ascribed to the yeast endogenous Fps1p [39,40], and phosphorylation affects Fps1p transport activity [25]. Phosphorylation of plant and mammalian aquaporins was also shown to modulate transport function as well as localization [41-43] and GmNOD26 of *Glycine max *is phosphorylated during specific developmental stages [44]. Strikingly, all isoforms that need to be truncated for functional expression in yeast contain a consensus Mitogen Activated Protein Kinase (MAPK) phosphorylation site [32] consisting of a Pro-X-Ser/Thr-Pro motif in their N-terminus. This motif is missing in other NIPII homologs. The conservation of this site (Table 1) suggests that phosphorylation is required for functional expression and that phosphorylation may have a regulatory role in plants. However, deletion of the conserved MAPK site in the case of AtNIP6;1 (*Atnip6;1*Δ_2–29_) and subsequent expression in yeast did not result in As(III) transport activity (Figures 2 and 3). Hence, the phosphorylation site itself is not identical with a potential inhibitory domain.

**Table 1:** N-terminal sequences of various plant NIPII isoforms containing a conserved MAPK phosphorylation site

Plant species	TC/EST/gene identifier	phosphorylation motif
*Allium cepa*	TC2332	A S A P A **T **P G **T **P G G P
*Gossypium spp*.	TC27968	A S A P A **T **P G **T **P G G P
*Lycopersicon esculentum*	TC174574	I S A P A **T **P G **T **P T P L
*Nicotiana tabacum*	TC8084	V S A P A **T **P G **T **P T P L
*Solanum tuberosum*	TC136541	I S A P A **T **P G **T **P T P L
*Lactuca sativa*	TC12433	V S A P A **T **P G **T **P G G P
*Populus spp*.	TC73227	V T A P N **T **P G **T **P G G P
*Populus spp*.	TC48087	V S A P N **T **P G **T **P G G P
*Aquilegia formosa *x *pubescence*	TC13964	P S **T **P A **T **P G **T **P G A P
*Pinus spp*.	TC61474	Q S I P T **T **P G **T **P G A P
*Saccharum officinarum*	TC64913	G S A P A **T **P G **T **P A P L
*Sorghum bicolor*	TC94800	G S A P A **T **P G **T **P A P L
*Triticium aestivum*	TC269691	L S A P A **T **P G **T **P A P L
*Brassica napus*	CD813862	S **T **P A T T P G **T **P G A P
*Petunia hybrida*	CV298254	P S **T **P A **T **P G **T **P G G P
*Vitis vinifera*	CU459322	P S **T **P V **T **P S **T **P G A P
*Atriplex numeria*	AnNIP1;1	L S V P P **T **P E G T P A P
*Atriplex numeria*	AnNIP1;2	E V S L S V P P **T **P E G T
*Lotus japonicus*	LjNIP5;1	T S M P A **T **P E **T **P G G P
*Lotus japonicus*	LjNIP6;1	P S **T **P A T T P G H T P G
*Medicago truncatula*	MtNIP4	P S T P A **T **P G **T **P G V P
*Zea mays*	ZmNIP3;1	G S A P A **T **P G **T **P A P L
*Arabidopsis thaliana*	AtNIP5;1	P P **T **P G **T **P G **T **P G G P
*Arabidopsis thaliana*	AtNIP6;1	S **T **P A T T P G **T **P G A P

Based on the amino acids forming the selectivity filter, the NIP subfamily of plant aquaporins has been divided into two subgroups, NIPI and NIPII [30-33]. According to this classification, NIPI group homologs are characterized by the consensus motif W I/V A/G R (Table 2). The NIPII group was initially defined for homologs characterized by the consensus motif A I/V A/G R. Later, OsNIP2;1, OsNIP2;2 and OsNIP3;2 were also placed in the NIPII subgroup [32], despite having a slightly different pattern of amino acids in their selective filter. Furthermore, the amino acid motif in the constriction region of LjNIP6;1 is distinct from the aforementioned examples but similar to MtNIP3 and MtNIP4 from *Medicago trunculata *(Table 2), all of which group to NIPII in our phylogenetic analysis (Figure 4). However, common to all NIPII isoforms, is a small hydrophilic amino acid residue at the first position, where animal, microbial and other plant NIPs have either an aromatic F or a W. The division into NIPI and NIPII also appears to be a valuable grouping from a functional perspective, as OsNIP2;1, OsNIP2;2 and OsNIP3;2 also proved to channel As(III) and Sb(III) similar to other NIPII proteins tested in this study. However, the diverse patterns of amino acids in the selective filters of all As(III) and Sb(III) transporting aquaporin homologs from various organisms (Table 2) strongly suggests that other features must be important for the selectivity of these channels. Interestingly, NIP isoforms that facilitate the diffusion of other hydroxylated metalloids such as B(OH)_3 _[21] and Si(OH)_4 _[22] also belong to the NIPII subgroup (Figure 4). Thus, accumulating evidence suggests that in plants, NIPII aquaporins are metalloid channels. In contrast, none of the isoforms of the NIPI subgroup tested here mediated As(III) transport. Members of the NIPI subgroup are expressed upon symbiosis with rhizobium and localized to the peribacteroid membrane in legume plants [44-46] and in plants interacting with mycorrhizal fungi [47]. Recently, *AtNIP2;1*, a member of the NIPI subgroup in *Arabidopsis *was shown to be induced at transcriptional level upon exposure to anoxic conditions [48]. When heterologously expressed in oocytes, AtNIP2;1 significantly increased the permeability for lactic acid, whereas only a minor permeability for water and glycerol, the 'conventional' aquaporin substrates, was recorded [48].

**Table 2:** As(III) and Sb(III) transporting aquaporin homologs, comparison of the constriction region and summary of transport properties

AQP	R1	R2	R3	R4	As(III) influx	As(III) efflux	Sb(III) influx	Reference
AtNIP1;1	W	V	A	R	-	-	-	This study
AtNIP2;1	W	V	A	R	-	-	-	This study
OsNIP1;1	W	V	A	R	-	-	-	This study
								
AtNIP5;1	A	I	G	R	••	•••	•	This study
AtNIP6;1	A	I	A	R	••	•••	•	This study
AtNIP7;1	A	V	G	R	•	-	•••	This study
OsNIP2:1	G	S	G	R	•••	•••	•••	This study
OsNIP2:2	G	S	G	R	•	-	•	This study
OsNIP3;2	A	A	A	R	•••	••	••	This study
LjNIP5;1	A	I	G	R	••	•	••	This study
LjNIP6;1	T	I	A	R	•••	•••	•••	This study
rAQP9	F	S	C	R	••• /+	• /nd	••• /+	This study /[26]
ScFps1	W	N	T	R	• /+	- /nd	• /+	This study /[24]
hAQP9	F	A	C	R	+	nd	nd	[53]
hAQP7	F	G	Y	R	+	nd	nd	[53]
EcGlpF	W	G	F	R	+	nd	+	[65,66]
SmAqpS	T	A	S	V	+	+	+	[56]

Aquaporin (AQP) homologs tested for transport of arsenite and antimonite. The tetrad of amino acids from transmembrane helix 2 (R1), transmembrane helix 5 (R2), and 2 residues from loop E (R3 and R4) that constitutes the selective filter are displayed. The abilities to channel the influx or efflux of arsenite or influx of antimonite of the tested aquaporins in various growth and transport assays are ranked (nd, not determined; +, transport; -, no transport and from • weakest to ••• best transport).

Glycerol has often been used as a substrate to monitor NIP channel activity [27,28,44,49]. Recent studies have shown that As(III) and Sb(III) exist as hydroxylated As(OH)_3 _and Sb(OH)_3 _at neutral pH [50,51]. Moreover, As(OH)_3 _and Sb(OH)_3 _show very strong structural similarity both to each other and to glycerol [50]. Hence, it was proposed that As(OH)_3 _and Sb(OH)_3 _transit through aquaglyceroporins through the same path as glycerol [50]. Similar conclusions could be drawn for NIPs in plants. However, in plants there is no direct evidence for a physiological role of glycerol transport through NIPs. Instead, metalloids may in fact represent the primary substrate of NIPII homologs. Notably, experimental data show that permeability for glycerol does not always coincide with the transport capacity for As(III) and Sb(III): (i) AtNIP1;1 transports glycerol [28] but does not seem to transport either As(III) or Sb(III) (this work); and (ii) OsNIP2;1 is shown here to be permeable for As(III) but is impermeable for glycerol [22]. The variability in the constriction region [33] along with other structural features of the channel path of NIPs seem to play a role in the discrimination between structurally highly similar solutes such as As(III), Sb(III) and glycerol. Interestingly, our phylogenetic analysis with rAQP9 as an out-group suggests that an ancestral NIP was capable of metalloid transport and that this capacity was lost in NIPI proteins (Figure 4). Nevertheless, this inference will require rigorous testing of more NIPs for the presence or absence of metalloid transport.

Due to the close structural similarity between As(OH)_3 _and Sb(OH)_3_, it was proposed that any difference observed in the toxicological properties of these metalloids must be related to processes other than cellular uptake [50]. However, in our study, expression of *AtNIP7;1 *only had a marginal effect on yeast growth on medium containing As(III), whereas on medium containing Sb(III), growth of the same transformants was strongly impaired (Figure 1). For yeast expressing *Atnip5;1*Δ_2–67 _and *Atnip6;1*Δ_2–69_, the opposite effects were seen with high sensitivity towards As(III) but only a mild increase in sensitivity towards Sb(III) (Figure 1). Thus, despite the high structural similarity of the two metalloids, our data demonstrate that NIPs can to some extent discriminate between As(III) and Sb(III) for selective transport.

OsNIP2;1 was originally identified as a Si(OH)_4 _specific channel without significant permeability for glycerol [22], and AtNIP5;1 was shown to be crucial for the uptake of B(OH)_3 _[21]. AtNIP5;1 and OsNIP2;1 are localized to the distal plasma membrane domain of the endodermis in *Arabidopsis *roots [21], and endodermis and exodermis of rice roots [22]. These cell layers represent a border for apoplastic transport. Nutrients taken up into the shoot via the vascular system have to enter the symplast by transport across the plasma membrane. Here we show that OsNIP2;1 and AtNIP5;1 are also permeable for the toxic metalloids As(III) and Sb(III). From a physiological point of view, the permeability for both beneficial (silicon) and essential (boron) nutrients as well as toxic substrates could pose a major problem for the plant; in the presence of arsenic in the soil solution, the expressed channels could be responsible for the uptake of toxic As(III). This would be problematic for rice, in particular, as rice roots grow in low oxygen soils, favoring the formation of the reduced form As(III). Hence, under conditions of low Si in the soil, leading to induction of *OsNIP2;1*, and under concomitant presence of As(III), rice roots are likely to take up As(III) via OsNIP2;1. Our data indicate that under physiologically relevant concentrations, Si(OH)_4 _does not compete with As(III) for uptake through OsNIP2;1, suggesting that OsNIP2;1 has a high capacity for the transport of both metalloids. It will be important to investigate if OsNIP2;1 and AtNIP5;1 are differentially regulated by As(III) versus Si(OH)_4 _and B(OH)_3 _in rice and *Arabidopsis*, respectively.

Transport of As(III) through NIPs and translocation to the shoot is potentially interesting for strategies of phytoremediation and the removal of arsenic from contaminated sites. In rice, uptake and translocation of Si(OH)_4 _is achieved by the concerted action of OsNIP2;1 and OsLsi2, the latter displaying homology to plant citrate transporters and to the *Escherichia coli *ArsB As(III) efflux transporter [22,52]. Rice Lsi2 is localized to the proximal plasma membrane domains of endodermis and exodermis cells. Si(OH)_4_, entering the cells via OsNIP2;1 is actively transported out of the cell and into the apoplastic continuum of the water transporting xylem elements for long-distance transport to the shoot [22]. However, expression of *OsLsi2 *or its closest homologs *Os10g0547500 *and *At1g02260 *did not affect yeast growth in the presence of As(III) or As(V) (data not shown). Although our data do not indicate that OsLsi2 and its homologs transport As(III) in yeast, it will be important to investigate this issue in plants.

Mammalian AQP9 was earlier shown to transport As(III) when expressed in yeast and *Xenopus *oocytes [26,53,54]. Interestingly, mono-methylated As(III), which is formed inside liver cells, was transported three times more efficiently than inorganic As(III), which led us to suggest that AQP9 could serve a function in arsenic detoxification [20]. Here, rAQP9 was very efficient in the uptake of As(III) but less effective in releasing the As(III) produced by yeast cells through the reduction of As(V). In contrast, NIP isoforms investigated here were less effective than rAQP9 in the uptake of As(III), but proved superior in supporting yeast growth in the presence of As(V). Plant cells primarily take up the oxidized form As(V) via high affinity phosphate transporters at the plasma membrane. It was recently reported that roots of rice and tomato plants have the capacity to efflux As(III) at high rate, when provided with As(V) in the growth medium [55]. The efficiency of some NIPIIs in extruding As(III) seen here supports the hypothesis that NIPII homologs may play a physiological role in arsenic detoxification. Such a role has been ascribed to the aquaglyceroporin AqpS from the legume symbiont *Sinorhizobium meliloti *[56]. Although this work demonstrates that specific plant aquaporins facilitate As(III) and Sb(III) transport, more work is needed to understand the role of As(III) transport through NIPII homologs in planta and we are currently putting efforts into this.

In conclusion, we demonstrate that specific plant NIPs transport As(III) and Sb(III) across cell membranes. Since the influx and efflux of toxic compounds is intimately linked to toxicity and detoxification, the identification of the proteins involved in this process provides a first important step towards the development of strategies for improving tolerance and remediation by plants.

## Methods

### Yeast strains and growth

W303-1A derived yeast strains (*MATa ura3-1 leu2-3/112 trp1-1 his3-11/15 ade2-1 can1-100 GAL SUC2 mal0 fps1Δ::LEU2 *[57] and *MATa ura3-1 leu2-3/112 trp1-1 his3-11/15 ade2-1 can1-100 GAL SUC2 mal0 fps1Δ::LEU2 ycf1Δ::loxP acr3Δ::loxP-kanMX-loxP *[58]) were used in this study. Yeast cells were grown on synthetic medium containing 2% glucose (SD) or 2% galactose (SG), 50 mM succinic acid/Tris base, pH 5.5, 0.7% yeast nitrogen base w/o amino acids (Difco) and supplemented with 0.003% each tryptophane, leucine, adenine and histidine according to the auxothrophic requirements of specific strains. Yeast cells were diluted in sterile water to an A_600 _of 0.01 (or various dilutions as indicated), and 10 μl were spotted on solid SG-medium containing various concentrations of sodium arsenite (Sigma), sodium arsenate (Aldrich) or potassium antimonyl tartrate (Hopkins and Williams LTD) as indicated. After 2 to 6 days of incubation at 30°C, differences in growth were scored. Data were duplicated in at least two independent experiments with consistent results.

### Cloning of plant aquaporins

The open reading frames of plant sequences were amplified by polymerase chain reaction (PCR) using specific primers (Table 3). The resulting PCR products were cloned into the yeast expression vector pYES2.1 (Invitrogen) and verified by DNA sequencing. Some of the open reading frames were cloned using a uracil-excision based cloning as described by Nour-Eldin et al. [59]. Oligonucleotides (Table 3) matching the EST sequences AV764898, AV419109 and BP048179 were used in a PCR on single-stranded cDNA prepared from five-week-old *L. japonicus *seedlings. Two independent PCRs for each aquaporin were performed. The PCR products of the expected size were sub-cloned and three clones were sequenced for each gene. Two full-length sequences were obtained and submitted to GenBank under EU294214 and EU294215[37] for *LjNIP5;1 *and *LjNIP6;1*, respectively.

**Table 3:** GenBank accessions and primers used for the cloning of the various plant cDNAs

Name used in this work	GenBank accession number to protein sequence	GenBank accession number to nucleotide sequence	Forward and reverse primer for the cloning of the genes
AtNIP1;1	CAA16760	Y07625	Forward 5' ATGGCGGATATCTCGGGAAAC 3' Reverse 5' TCAAGTGCTACCGATTCTCACG 3'
*Atnip1;1Δ*_2–44_			Forward 5' ATGTCTCTACTCTCAGTCTCTGTCCCT 3' Reverse 5' TCAAGTGCTACCGATTCTCACG 3'
AtNIP2;1	T02327	AJ276475	Forward 5' ATGGATGACATATCAGTGAGCA 3' Reverse 5' TCACAGAGGAAGATCGGTAAC 3'
*Atnip2;1Δ*_2–37_			Forward 5' ATGTCCCCTCCTTTGCTC 3' Reverse 5' TCACAGAGGAAGATCGGTAAC 3'
AtNIP5;1	T04053	AY087560	Forward 5' ATGGCTCCACCGGAGGCTGA 3' Reverse 5' TTAACGACGAAAGCTCCTAACCGG 3'
*Atnip5;1Δ*_2–67_			Forward 5' ATGGATTTTCCCTCTCCTGATGTCTC 3' Reverse 5' TTAACGACGAAAGCTCCTAACCGG 3'
AtNIP6;1	AAF14664	BT020229	Forward 5' ATGGATCATGAGGAAATTC 3' Reverse 5' TCATCTTCTGAAGCTCCTC 3'
*Atnip6;1Δ*_2–29_			Forward 5' ATGGGGAAGAGGAATGGACAC3' Reverse 5' TCATCTTCTGAAGCTCCTC 3'
*Atnip6;1Δ*_2–69_			Forward 5' ATGTCTCTCCCTCCCCCTAA 3' Reverse 5' TCATCTTCTGAAGCTCCTC 3'
AtNIP7;1	AAF30303	AY087797	Forward 5' ATGAATGGTGAGGCACGGTCA 3' Reverse 5' TTAACGTAAAAGTGAAGAAACGGAAGG 3'
*Atnip7;1Δ*_2–35_			Forward 5' ATGCTCCCCTATGATATAGATCTCAATC 3' Reverse 5' TTAACGTAAAAGTGAAGAAACGGAAGG 3'
LjNIP5;1	EU294214	ABY19373	Forward 5' ATGCCTCCGCTGTGGAAGAA 3' Reverse 5' CTAGCGACGGAAGCTCCTAACC 3'
*Ljnip5;1Δ*_2–59_			Forward 5' ATGGATTTCTCTGCTGGTATTGG 3' Reverse 5' CTAGCGACGGAAGCTCCTAACC 3'
LjNIP6;1	EU294215	ABY19374	Forward 5' ATGGACAACAATGAGGACATTCC 3' Reverse 5' TCACCTTCTGAAGCTGGGGT 3'
*Ljnip6;1Δ*_2–72_			Forward 5' ATGTCATTGCCATCCCCTCCT 3' Reverse 5' TCACCTTCTGAAGCTGGGGT 3'
OsNIP1;1	Q40746	D17443	Forward 5'GGCTTAAUATGGCAGGAGGTGACAACAA3' Reverse 5'GGTTTAAUTTAGGTGGAGGAGTTCATCCG 3'
OsNIP2;1	Q6Z2T3	AB222272	Forward 5'GGCTTAAUATGGCGAGCAACAACTCCAGA 3' Reverse 5'GGTTTAAUTCACACTTGAATGTTCTCCATCTCG 3'
OsNIP2;2	Q67WJ8	AP003569	Forward 5'GGCTTAAUATGGCATCGACGACAGCG 3' Reverse 5'GGTTTAAUTTATACGTTGTCGAACTCGTCGG 3'
OsNIP3;2	Q7EYH7	AP004461	Forward 5'GGCTTAAUATGGAAGGGGGCAAGATG 3' Reverse 5'GGTTTAAUCTACAGCTTGATTGCAAAATAAAAC3'
At1g02260	AAY56454	BT023463	Forward 5'GGCTTAAUATGGCAATGGCTCCTGTAA 3' Reverse 5'GGTTTAAUTTACTTGATAAGGAAGAGACCAATC3'
OsLsi2	NP_001048691	NM_001055226	Forward 5'GGCTTAAUATGAGTGAGCTTGCGTCGG 3' Reverse 5'GGTTTAAUTCAGATCTTGCCGATGAGGG 3'
Os10g0547500	NP_001065297	NM_001071829	Forward 5'GGCTTAAUATGGCACTGGCGTCGCTG 3' Reverse 5'GGTTTAAUCTAGATGTTGATCTTGCCGATGAGA 3'

### Membrane protein extraction and Western blotting

*S. cerevisiae *(Δ*fps1 *and Δ*fps1 *Δ*acr3 *Δ*ycf1*) expressing individual plant genes or transformed with the empty vector pYES2.1 were pre-cultured over night in selective medium and expression was induced in a subsequent over night culture in 1% yeast extract (Difco), 2% peptone (Difco) and 2% galactose. Cells were harvested, re-suspended in homogenization buffer (220 mM Tris/HCl pH 7.5, 42 mM EDTA pH 8, 42.5% glycerol, 10 μM ATP and proteinase inhibitor cocktail (Roche)) and broken using acid washed glass beads (Sigma). After removal of cell debris by centrifugation (8000 *g *for 15 minutes), the microsomal membrane fractions were collected at 100,000 *g *for 1 hour. Pellets were resuspended and homogenized in resuspension buffer (20% glycerol, 100 mM Tris/HCl pH 7.5, 1 mM EDTA pH 8, 1 mM DTT, and proteinase inhibitor cocktail). Protein concentrations were determined according to Bradford using the assay from Bio-Rad with BSA as standard. We separated 20 μg of membrane protein on SDS-polyacrylamid gels (4% stacking gel; 12% separation gel) and transferred onto nitrocellulose membranes (Bio-Rad) by electroblotting. Membranes were blocked in 2% milk powder in TBS. Primary and secondary antibodies were diluted in TBS at 1:500 (anti-NIP6;1 antibody) and 1:10,000 (goat anti-rabbit IgG-AP conjugate, Bio-Rad), respectively. Detection was performed using NBT/BCIP (Promega). Coomassie stained gels were routinely run as loading control.

### Arsenic transport assays

Arsenic transport assays were performed essentially as described previously [24]. Briefly, exponentially growing yeast cells in SG medium were exposed to sodium arsenite. Cells were collected at the time points indicated in the figures, washed in ice-cold water and pelleted by centrifugation. The cell pellets were resuspended in water, boiled, centrifuged and the supernatants were collected. The arsenic content of each sample was determined using a graphite furnace atomic absorption spectrometer (SIMAA 6000; Perkin-Elmer) as described previously [60]. For competition assays, equimolar or 10-fold molar excess of Si(OH)_4 _or B(OH)_3 _was added together with the As(III). Samples for arsenic content were collected and analyzed as above. All transport assays were performed at least three times and the values are given with SD.

### Alignment and phylogenetic analyses

33 sequences corresponding to plant NIP homologs plus rat AQP9 (out-group) were aligned in MAFFT 6.240 [61] and analyzed phylogenetically in MrBayes 3.0 [62] (Bayesian analysis: 5 million generations, 1 tree sampled every 5000 generations, 8 MCMCMC chains and a 50% burn-in) and PAUP 4.0b10 [63] (parsimony analysis: 10,000 random addition sequences with 10 trees held per step and TBR branch swapping with at most 25 trees saved per round; branch support was estimated through 25,000 jackknife replicates). The final alignment consisted of 34 sequences and 315 amino acid residues, 212 (67%) of which were parsimony informative. The 50% majority-rule consensus phylogram computed from the resulting 2500 trees of the Bayesian analysis is shown in Figure 4. In the parsimony analysis, one of the most parsimonious trees of length 1644 was recovered (CI = 0.6150, RI = 0.6941). The support values from the jackknife analysis are superimposed onto the Bayesian tree in Figure 4[64].

## Authors' contributions

GPB cloned the plant aquaporin isoforms, performed most of the growth assays and wrote the first draft of the manuscript. MT performed some of the growth experiments, conducted the uptake assays and participated in writing the manuscript. MDS performed the Western-blotting analysis and participated in writing the manuscript. HRN performed the phylogenetic analyses. AW performed the atomic absorption analysis. MJT and TPJ initiated the project and finalized writing the manuscript. All authors read and approved the final manuscript.
